# The Estimation of Active Social Network Size of the Iranian Population

**DOI:** 10.5539/gjhs.v5n4p217

**Published:** 2013-06-17

**Authors:** Azam Rastegari, Saiedeh Haji-Maghsoudi, Aliakbar Haghdoost, Mohsen Shatti, Termeh Tarjoman, Mohammad Reza Baneshi

**Affiliations:** 1 Regional Knowledge Hub for HIV/AIDS Surveillance, Kerman University of Medical Sciences, Kerman, Iran; 2 Medical Student Research Committee, Kerman University of Medical Sciences, Kerman, Iran; 3 Social Determinant of Health Research Center, Institute for Futures Studies in Health, Kerman University of Medical Sciences, Kerman, Iran; 4 Candidate of Epidemiology, Department of Biostatistics and Epidemiology, School of Public Health, Tehran University of Medical Sciences, Tehran, Iran; 5 Expert of Mental and Social Health, Addiction Dep. Health Deputy of Ministry of Health and Medical Education, Tehran, Iran; 6 Research Center for Modeling in Health, Institute for Futures Studies in Health, Kerman University of Medical Sciences, Kerman, Iran

**Keywords:** network size, network scale up, size estimation, Iran

## Abstract

**Objectives:**

The size of active network (C) of Iranian population is a very important parameter to estimate the size of unknown population using Network Scale Up (NSU) technique. However, there is little information about this parameter not only in Iran but also in other countries in Middle East region. Based on these needs, the aim of this paper is to estimate C for the Iranian population.

**Methods:**

Based on available national statistics, 23 reference groups, with known population sizes were selected. Using multistage sampling method, 7454 individuals were recruited randomly around the country. We asked from our samples how many people they knew from each of the reference groups. Using NSU formulae, we maximized the goodness of fit of our estimation about the size of the reference groups by fitting the best C. However, the final C was set by excluding some of the reference groups with no added information; these inappropriate groups were selected by two techniques; regression, and ratio based approaches.

**Results:**

Applying regression and ratio based approaches the estimated C was 308 and 380 respectively. The Pearson correlation coefficient between the real and estimated size of reference groups (based on our C) in both methods was above 0.95. However, results of ratio based had better performance. We saw that the network of males, singles, younger age groups, and those with higher education was larger than those in other groups.

**Conclusion:**

It seems that C in Iran is higher than that in developed countries, possibly because of its social structure. Because of cultural and social similarities in Middle East courtiers, C in other countries also might be higher than that in developed countries.

## 1. Introduction

In many situations, we wish to know the number of people who share common features. When the characteristics of interest is not sensitive, such as the number of car accidents, we can get an accurate figure from national records. However, when the characteristics is stigmatized (i.e. the number of female sex workers (FSWs)), formal statistics might not be available. This is the case especially in majority of Islamic countries where these behaviors are considered against prevailing Islamic values (Kadushin et al., 2006; Paniotto et al., 2009).

In the case of hidden groups, the application of direct size estimation methods such as surveys is not feasible. Therefore, indirect size estimation methods are required. Among indirect methods, Network Scale Up (NSU) has practical appeals, as no direct contact with members of hidden groups is needed. Furthermore, it is possible to estimate the size of several hidden groups in a single study (Salganik et al., 2011; Killworth et al., 1998; Paniotto et al., 2009).

The NSU method depends on the active network size of population (shown by C). The idea behind the NSU method is that the proportion of individuals belonging to a sub-population in the network of a representative sample has a direct association with the real size of that sub-population in the general population.

Network estimation studies have been mostly performed in USA so far (Johnsen et al., 1995; Killworth et al., 1998; Kadushin et al., 2006). In 2009, Ukraine performed a similar study to estimate size of key groups at high risk of HIV (Paniotto et al., 2009). In recent years, size estimation studies have been carried out in East Asia in countries such as China, Japan, and Thailand (Bao et al., 2012; UNAIDS, 2012; Ezoe et al., 2012). In addition some studies have conducted in Brazil, Rwanda, and Moldova (Salganik et al., 2011; IHDPC, 2012; UNAIDS, 2012). There is limited data on the size of hidden groups in developing countries such as Iran, which is essential to policy makers. No similar study in the Middle East and North Africa (MENA) region has been found in our extensive search.

To be able to estimate the size of such groups using the NSU method, the estimation of C is a prime. To our best knowledge, only one size estimation study has been carried out in an Iranian province (by the research team of this study); having recruited 500 respondents, authors found that the average active network size among 18 to 48 year-old males was about 303. However, because of the scope of the sample frame of the study, this figure might not be generalized to the whole country. Therefore, we designed a national-wide study to estimate the network size (C) of Iranian population, and to identify potential demographic factors that might influence it.

## 2. Methods

Iran is comprised of 31 provinces. At the first step, based on the geographical location and ethnicity of people, we divided the whole country into 15 stratums. From each stratum, we selected one province. In total, we recruited 7454 respondents from 15 provinces proportional to their size. In each province, samples were selected from the capital and one of the large cities (75% from the capitals and 25% from large cities).

In this study, we selected 23 reference groups with known population size (shown by e) from independent data sources (Table 1). For the first step, we asked our samples the number of people they knew in their active network that belonged to each reference group (shown by m). The definition of “know” was “people whom you know and who know you, in appearance or by name, with whom you can interact, if needed, and with whom you have contacted over the last two years personally, or by telephone or e-mail” (Midanik & Greenfield, 2003; Pridemore et al., 2005; Shokoohi et al., 2010). These numbers are associated with C and the real size of every reference group (e). Then we estimated C following different strategies.

**Table 1:** List of reference groups, organization provided the data, real size, and estimated size from regression and ratio based strategies

Ref NO	Reference group	Organization provided the data	Real size (proportion)	Regression		Ratio	
				Estimate	Ratio	Estimate	Ratio
1	Study in the first year of elementary school in past year	Ministry of Training & Education	1169974 (0.0157)	-	-	-	-
2	Work in elementary school in past year	Ministry of Training & Education	279948 (0.0037)	245511	1.14	303079	0.92
3	Graduated from high school in past year	Ministry of Training & Education	478423 (0.0064)	426732	1.43	362725	1.32
4	Married in past year	Vital statistics registration	859288 (0.0115)	-	-	-	-
5	Divorced in past year	Vital statistics registration	137200 (0.0018)	96370	1.42	118967	1.15
6	NVD in past year	Ministry of Health	643723 (0.0086)	-	-	-	-
7	C/S in past year	Ministry of Health	611456 (0.0082)	-	-	-	-
8	Thalassemia	Ministry of Health	21817 (0.0003)	80581	0.27	-	-
9	Absolute blind	Welfare organization	19336 (0.0003)	51875	0.37	-	-
10	Death caused by vehicle accident	Ministry of Health	23249 (0.0003)	-	-	-	-
11	First name ”Hamed”	Vital statistics registration	206942 (0.0028)	221050	0.94	272881	0.76
12	First name ”Shahin”	Vital statistics registration	37158 (0.0005)	106018	0.35	-	-
13	First name ”Afshin”	Vital statistics registration	62704 (0.0008)	138202	0.45	-	-
14	First name ”Abolfazl”	Vital statistics registration	414187 (0.0055)	270553	1.53	333992	1.24
15	First name ”Eisa ”	Vital statistics registration	119784 (0.0016)	120515	0.99	148773	0.81
16	First name ”Sara”	Vital statistics registration	249592 (0.0033)	214012	1.17	264193	0.94
17	First name ”Mandana”	Vital statistics registration	15231 (0.0002)	66426	0.23	-	-
18	First name ”Hadith”	Vital statistics registration	64743 (0.0009)	117668	0.55	-	-
19	First name ”Nadereh”	Vital statistics registration	11041 (0.0001)	38274	0.29	-	-
20	First name ”Marjan”	Vital statistics registration	81321 (0.0011)	126894	0.64	156648	0.52
21	Epilepsy	Ministry of Health	73800 (0.001)	85747	0.86	105853	0.70
22	Participate in entrance national university exam	Sanjesh organisation	610018 (0.0082)	426732	1.43	526792	1.16
23	Freshman university student in past year	Sanjesh organisation	252786 (0.0034)	251073	1.01	309945	0.82

### 2.1 Use of all Reference Groups

The basic equation for estimation of C is e/t = m/C. This formula has been extended to derive maximum likelihood estimation of C, and to include information of more than one reference groups. For each of 7454 subject using the equation 1, we estimated national C_i_. Its associated Standard Error (SE) was estimated applying formula 2:


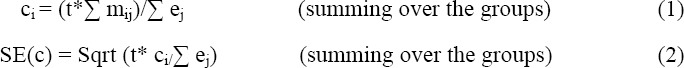


Here, indices i and j stand for the respondent and reference group respectively. Final C was the average of C_i_ (Killworth et al., 2003; Paniotto et al., 2009; Snidero et al., 2009). Estimated C can be used to back calculate the size of reference groups (formula 3).


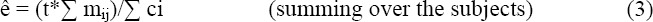


As a sensitivity analysis, we calculated network size at provincial level as well. Average of province estimates was used as final statistics.

Formula 1 combines the information of all reference groups together to provide an accurate estimate of C. Some size estimation studies did not back calculate how accurate size of reference groups would be estimated. However, some of these groups do not have enough eligibility to be considered as an appropriate reference group. This is because respondents usually undercount the size of some of more prevalent, and overestimate that of some of less prevalent reference groups (known as recall bias) (Salganik et al., 2011; Killworth et al., 2003). A recent work in Ukraine followed a ratio based approach (explained later) (Paniotto et al., 2009). In this paper, we selected the eligible reference groups by two different approaches as follow.

### 2.2 Excluding Ineligible Groups via Regression Based Approach

Formula 1 implies that there is a linear association between prevalence of reference groups in the society (i.e. e/t) and average number of people respondents knew in each reference group (i.e. mean of m). However, there are evidences that this is not necessarily always the case, partially due to recall bias. Killworth et al. (2003) showed that mean m was associated with square root of e/t. To be able to use the standard formula that estimates C (i.e. formula 1), we applied a series of multilevel and regression analyses to exclude reference groups which does not fit in the linearity assumption. We fitted a regression line and calculated standardized DFBETA statistic (SDFBETA) for all reference groups. SDFBETA measures the changes in regression coefficient per deletion of each of the reference groups. The reference group with the highest SDFBETA was excluded to justify the linearity. The whole process was continued in an iterative fashion to remove all reference groups with SDFBETA higher than 3/√n (where n is sample size reference groups).

### 2.3 Excluding Ineligible Groups via Ratio Based

In the ratio based approach, we first used the information from all reference groups. Combining the information via formula 1, C was estimated. This was followed by the back calculation of the sizes of all reference groups applying formula 3. Then, for each reference group, we divided the real to the estimated size. Ratios close to one (range 0.5 to 1.5) were considered as ideal. We deleted the reference group with the worst ratio. A new C was re-estimated using the rest of the reference groups. The new C was then used to back-calculate size of the reference groups. The whole process was applied iteratively until all ratios stayed between 0.5 and 1.5 (Killworth et al., 1998; McCarty et al., 2001; Killworth et al., 2003; Paniotto et al., 2009; Snidero et al., 2009)

To identify factors that might influence C we applied a random effects model. The effect of gender, age, and education was considered fixed. Province was entered into the model as a random factor. All analyses were performed using stata version 11 and Microsoft Excel software.

### 2.4 Comparison and Validation of Performance of C Values

We compared performance of different C values in terms of the correlation between real and estimated size of reference groups, Mean Square of Error (MSE), and Positive and Negative Mean Absolute Bias (PMAB and NMAB). To estimate MSE, we averaged the square difference between real and predicted sizes divided by estimated size. To estimate PMAB and NMAB, we calculated the ratio of real to estimated sizes. Mean of values above 1 was considered as PMAB. NMAB was estimated as the mean of values less than 1.

We also checked the ability of C values derived in estimation of size of reference groups which did not contribute in estimation of network size (i.e. ineligible reference groups).

As another measure of agreement, we fitted a regression through to the origin line in which predicted and real size of reference groups were used as dependent and independent variables. Clearly a slope close to one indicates agreement between predicted and real values.

To validate our estimates, we divided the data into two parts. Following strategies explained above, C was estimated in each half of data independently.

## 3. Results

Our sample comprised of 3584 (48.2%) males (out of 7454 respondents). The mean (SD) age for male and female samples were 30.79 (11.27) and 30.80 (10.11) respectively; the youngest and oldest subjects were 18 and 87 years old respectively. Nearly 40% of samples were single, and around 57% of respondents had 12 or less years of formal education (diploma or less) (Table 2).

**Table 2:** Demographic characteristics of respondents

Variable	Sample size (proportion)
Gender	
Male	3584 (48.2)
Female	3853 (51.8)
Marital Status	
Single	3155 (42.3)
Married	3899 (52.3)
Divorce/Widow	295 (4)
Formal Education	
<12 years	4237 (56.9)
12-16 years	2847 (38.3)
> 16 years	358 (4.8)

### 3.1 Using all Reference Groups

**Figure 1:**
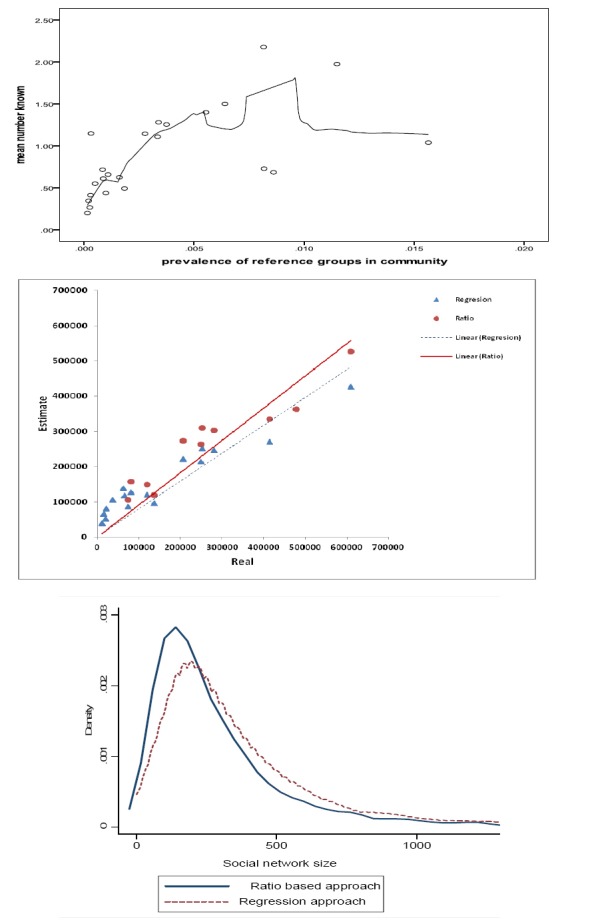
Scatter plot of the prevalence of reference groups versus mean number known by respondents (top panel), real versus predicted size from ratio and regression based methods (middle panel), and distribution of network size based on two method (bottom panel)

At the first step, we combined information of all 23 reference groups. C was estimated at 239. The correlation coefficient between the real and predicted size of reference groups was 0.60. This gave coefficient of determination of 0.36 indicating poor ability of derived C in prediction of size of reference groups. We plotted prevalence of reference groups in the community versus mean number known by respondents (Figure 1 top panel). Lowes smoother confirms poor linear fit. Although respondents were able to answer the questions in a reasonable way, we have seen that respondents undercount the size of some of more prevalent, and overestimate that of some of less prevalent reference groups (recall bias). For example, more and less prevalent groups were ‘students at first year of elementary school’ and ‘death due to car accident in the last year’ with prevalence 1.6% and 0.03% respectively. However, mean people known by respondents were 1.04 and 1.12 respectively. In addition, MSE was 216780. PMAB and NMAB were estimated at 2.34 and 0.48 indicating high bias in back estimation of size of known groups. The slope (SE) of the regression line was 0.63 (0.10) which was significantly different with one (P-value=0.001)

As a sensitivity analysis we stratified our estimates based on province. C values ranged from 120 to 408, with a mean C of 248. Performance of this C (248) was fairly the same as national C (239). The slope (SE) of the regression line was 0.60 (0.09) indicating poor agreement between real and predicted sizes (P=0.0004). Table 3 provides performance of all C values. As these two C values produced poorest results, no further attention was paid to them.

**Table 3:** Comparison of performance of C values derived applying different strategies

Estimation method	Correlation of e and ê*(eligible groups)	Correlation of e and ê (ineligible groups)	MSE	PMAB	NMAB	Slope (SE)**
All reference groups (national) C= 239	0.60	Not applicable	216780	2.34	0.48	0.63 (0.10)
All reference groups (average of province estimates) C= 248	0.60	Not applicable	225674	2.20	0.47	0.60 (0.09)
Eligible groups via regression method C= 380	0.97	0.06	25422	1.28	0.54	0.79 (0.06)
Eligible groups via ratio method C= 308	0.95	0.63	13884	1.22	0.78	0.91 (0.06)

* The correlation between real (e) and estimated size (ê) of reference groups is the same as correlation between prevalence of reference groups in the community with average people known by respondents in each

** slope of regression through origin line of predicted versus real sizes

### 3.2 Excluding Ineligible Groups via Regression Based Method

Answer of 7454 respondents about the number of people they know in 23 reference groups was available. We structured the data in long format and applied multilevel random effects analysis, to explore the level of dependency of data. ICC was estimated at around 0.10 indicating very low correlation between measurements from the same subject. Due to a very low ICC, and to make our results comparable with other network estimation studies (Salganik et al., 2011; Paniotto et al., 2009), we deleted ineligible reference groups analysis grouped data. We therefore concentrated on prevalence of reference groups in the society and mean number of people respondents known in each reference group.

Analyzing all 23 reference groups, our results shows that the NSU assumption (linearity between e/t and mean m) is not satisfied by the data. To justify this assumption, we excluded six reference groups, one in turn. The minimum and maximum proportion of reference groups which were deleted, in the general population were 0.03% and 1.6%. Correlation for model containing eligible 17 reference groups was 0.97 (correlation of e/t versus mean m; which is the same as the correlation of e versus ê). However, correlation between real size of 6 ineligible reference groups and predicted sizes, based on C at 380, was as low as 0.06. The slope (SE) of the regression line was 0.79 (0.06) which was significantly different with one (P-value=0.003).

Based on information of 17 reference groups retained, the estimated C and its standard error for the whole Iranian population was 380 and 103.42 respectively.

In comparison with values derived from all reference groups (i.e. 239 and 248), MSE decreased sharply to 25422. PMAB and NMAB were improved.

The subgroup analysis showed that C in males were significantly higher than that in females (422 versus 341, P<0.001). In addition, we found a greater C among singles (398), young people (388), and more educated samples (448). In a random effects Poisson model, all these variables were significant. Furthermore, around 11% of the variation was associated with province (Table 4).

**Table 4:** Determination of factors influence network size of Iranian population derived from ratio and regression based methods

Variable	Ratio based			Regression based		
	Mean	SE	P-value	Mean	SE	P-value
Gender						
Male	344.72	6.28	<0.001	422.4	7.22	<0.001
Female	274.47	4.06		341.76	4.63	
Marital Status						
Single	335.69	6.26	<0.001	397.82	6.82	<0.001
Married	291.68	4.7		373.83	5.68	
Divorce/widow	255.62	17.19		314.78	18.04	
Age						
18-25	330.78	6.3	<0.001	388.36	6.78	<0.001
25-40	306.53	5.76		390.42	6.87	
>40	260.95	6.94		338.9	8.66	
Education						
<12 years	283.51	4.46	<0.001	351.83	5.15	<0.001
12-16 years	339.27	6.5		414.71	7.36	
>16 years	353.46	20.89		448.21	24.63	

### 3.3 Excluding Ineligible Reference Groups via Ratio Based Method

In total 12 reference groups were deleted, one in each step, as the ratio of estimated size to the real size was outside the range of 0.5 to 1.5. Final C was estimated at 308 with SE of 89.05 based on 11 groups. The real and estimated values of these 11 groups and calculated ratio are given in Table 1. The minimum and maximum ratio was 0.52 and 1.32. The correlation between real and estimated values was 0.95 (Figure 1 middle panel). Correlation between real size of 12 ineligible reference groups and predicted size, based on C at 308, was 0.63. MSE was even lower than regression approach (13884 vs. 25422). PMAB and NMAB were much closer to 1 indicating lower bias in back estimation of size of known groups. In addition, the slope (SE) of the regression line was 0.91 (0.06). The difference between this slope and one was not statistically significant (P-vale=0.17), indicating good agreement between predicted and real size of reference groups.

Classifying the C based on demographic variables, results were the same as the regression based. Network size for males was 25% larger than females (344 vs. 274). In addition, the network size of singles and young respondents was lower than other groups. Also, 11% of variation was explained by province.

### 3.4 Agreement between C Values Derived Applying Regression and Ratio Based Strategies

At individual level, the correlation between C values derived from regression and ratio based approaches were 0.94. Prediction of both approaches in estimation of size of reference groups were acceptable (Figure 1 middle panel). In addition, density graph of the estimated values at the population level were fairly similar (Figure 1 bottom panel).

### 3.5 Validation of Results

For validation of our results, we randomly split the data into two parts. Sample size in the first and second halves was 3000 and 4454. Applying ratio based method; results of the first and second half were similar. While analyzing whole data C was estimated at 308, figures correspond to first and second halves were 306 and 310 respectively. In addition, results of all three analyses were exactly the same in terms of reference groups excluded.

Following regression approach, C in the first and second halves was 376 and 461 respectively (C in whole data was 380). In addition, minor differences in terms of reference groups excluded were seen.

## 4. Discussion

### 4.1 Estimation of C

In this study we implemented four different methodologies to estimate C, and calculated four quite different C. We did not consider two of them (when all reference groups were considered) due to high bias in back estimation of size of reference groups. When we used all 23 reference groups C was estimated at 239. The correlation coefficient between real and predicted size of reference groups was 0.60. This gave coefficient of determination of 0.36 indicating poor ability of derived C in prediction of size of reference groups.

Analyzing eligible reference groups (17 in regression and 11 in ratio-based method), C was estimated at 380 and 308 respectively. We assessed internal validity of our results using different criterion. Back-calculating the size of reference groups, the correlation coefficient reached above 0.95 in both approaches. We have seen that ratio based method provided the lowest MSE, PMAB, and NMAB. Slope of predicted versus real regression, in ratio based approach, was not significantly different with zero. In addition, in data splitting analysis, estimates in first and second halves were fairly the same as analysing the whole data. Results of regression based method were much better than using all reference groups but was inferior to ratio based. C from ratio based approach was able to predict size of ineligible groups more accurately than that from regression based. We should emphasize that minor bias in estimation of size of reference groups was seen (Figure 1 middle panel). However, this is an issue in all size estimation studies (Killworth et al., 1998; Killworth et al., 1998). Besides that C value obtained through ratio approach showed acceptable performance in terms of prediction ability and internal validity.

The estimated C in different countries are quite different. It possibly shows the dependency of C to factors such as the cultural and social structures of communities. Our estimated C is more than that in the USA (108), and 286 and Ukraine (175) (Killworth et al., 1998; Paniotto et al., 2009). This difference might show that Iranian people have more personal links (Ghavamzadeh & Bahar, 1997). However, on top of the possible impact of cultural differences, the differences of used methodological in the studies might explain some of these differences (addressed later).

### 4.2 Size Estimation Studies in the World

Here some size studies which reported the size of network size of different communities are provided. Kadushin et al. (2006) recruited individuals aged 16 to 44 to estimate the size of heroin users. The estimated C was 55. This estimated C was very low which might be explained in different ways. First, to estimate C, six questions which were associated with heroin use were selected. These reference groups include number of those who were attacked, hit, or burglarized in the last year. Sensitivity of questions used in the estimation of C might partially explain low value obtained. One problem with this study was that ratio of estimated to real values were from 0.106 to 3.072. No further attempt was done to delete groups which were highly underestimated or overestimated to find a more reliable network size.

In another study, to estimate size of seroprevalence and rape in the US, 1554 subjects were recruited. Information of size of 29 reference and C was estimated at 286. Authors concluded that the high value obtained, relative to other studies, was mainly due to a larger number of reference groups used. Back calculating the size of reference groups, correlation between real and estimated sizes was 0.79. Authors have seen that two large reference groups were highly underestimated (due to recall bias). Omitting those two reference groups, correlation raised to 0.94 (based on C of 286). No new C value was derived without two large reference groups (Killworth et al., 1998).

In a similar study in Ukraine, 10,866 respondents were recruited; the estimated C was 175 (Killworth et al., 1998; Paniotto et al., 2009; Snidero et al., 2009). Recent size estimation studies in East Asia estimated size of network size of Japanese at 364 and Chinese at 310.

### 4.3 Impact of Methodology Implemented on Calculation of C

We have seen that selection of eligible reference groups via different approaches affects estimation of C. This is not surprising, however. In a study to estimate number of HIV sero+ cases, authors have applied six different strategies to estimate the average network size which produced 6 different values for the C; 97, 105, 105, 113, 117, and 399 (Killworth et al., 1998). We believe that the direct comparison of C values reported is not simple. This is because different methodologies were applied. In addition, reference groups used in different methods were not the same. For example, in the third and fifth approach, only information of 7 reference groups. The formula used in the third approach was not based on frequencies, but based on probability of knowing at least one member in reference groups. In none of the approaches was the back-calculation of reference groups performed. Applying these network size values, estimated size of sero+ subpopulation ranged 1.1 to 2.8 million. Authors concluded that second C (105) was not accurate as it led to estimate seropositives at 2.8 million. Also, sixth value (399) was not considered as being reliable due to large SE. The authors concluded that, based on other four estimates, the best guess for C was at 108.

### 4.4 Impact of Demographic Factors on C

We have checked whether demographic characteristics (gender, marriage, education, and age) of respondents affect C. We have seen a greater C in males, younger aged, educated, and single groups. Influence of these factors on network size has been confirmed in Chinese population as well (Bao et al., 2012).

The network size of men was greater than that in females. Although women's role in social activities has been increased in recent decades, still it seems that males have wider networks. Males have more freedom to communicate with others. In addition, women are usually communicating with less people but their links are stronger (MSc thesis submitted to Kerman University of Medical Sciences) (Halimi, 2011).

The network size of married couples was smaller than single subjects. It is not easy to explain this difference. From one side, it seems that a married person might have more contacts within his/her own network and the network of his/her spouse. However, from the other side, married people usually limit their communications within their friendship networks; this is particularly important in a family based community such as Iran. Smaller C among married subjects might be explained by the latter explanation.

Greater C among younger age-groups might be also due to the effect of marriage. By increasing age, more might get married, therefore their Cs decreases. In addition, young people usually create links with others much faster; however, their links are not strong and deep (Van Tilburg, 1998; Ajrouch et al., 2001).

Education was positively linked to the network size. The observed positive relationship between C and education level was also observed in other parts of the world (Roberts et al., 2008). One possible explanation which might partially justify our results is that usually education opens new opportunities for people to get high ranking jobs with more involvement in social affairs. For example, teachers, doctors, and academic staff might have more communications with others. In addition, these people are more approached by others because of their skills, knowledge, and social potions. Educated people can also communicate others through internet. All these factors might increase the network size of educated people. However, issues noted here should be addressed in other research.

### 4.5 Strengths and Weaknesses of Our Study

The estimation of network size needs careful planning and accurate information from known groups. One of the issues that affect the estimation of C is the number of reference groups. It has been suggested that, to get reliable results, the number of reference groups should be between 20 and 30. Selection of appropriate known groups was one of the most important considerations in our study. We have tried to select heterogeneous subgroups in different sizes and a wide prevalence ranges to have a diverse list of subgroups among the general population. The total size of known groups used in the present study was 16.1% of the whole population of the country. This number copes with recommended number in methodological considerations which is less than 20% (Paniotto et al., 2009; Shokoohi et al., 2010).

Another advantage of our study was that we recruited a large sample size. Relative to the sizes of other estimation studies, our sample size was very high. We also filled the questionnaire through face to face interviews at street-level. This is done to avoid attrition in sample size due to non-responses, and to get more accurate responses. Besides that, our experience confirmed that in Iran street-based interviews work better than telephone or home-based interviews (Comparison of three interview methods on response pattern to sensitive and non-sensitive questions [Under review]).

Having explained the advantages in this study, there were several limitations as well. Limited access to the exact number of subgroups populations is the major limitation in these kinds of studies. For example, multiple sources gathered information of the number of diabetic patients. However, their statistics did not match, and therefore we used the one which seemed more accurate. In addition, we have seen that province can explain a proportion of differences. Although our sample sizes in provinces were not high enough, as a sensitivity analysis, we reanalyzed the data at province level. We have seen that the network size varied from 120 to 408, at a mean of 248. This indicates that our results cannot be generalized to each province. Therefore, independent studies with adequate sample sizes are required to get results at the provincial level. This can be a topic for future studies.

We should highlight that in these methods, there is no gold standard. Therefore, assessment of external validity of results seems to be difficult. No similar study in MENA countries was found. Therefore, we are not able to compare this figure to countries with similar cultures to Iranian populations. We believe that other countries in the MENA region should implement similar studies to estimate network size, and then size of hidden groups. However, recent network estimation studies in East Asia reveal a network size of around 300.

In addition, we could not find any independent data source to cross-validate our findings. Results of our data splitting analysis, were promising. We hope that when using similar methodological approaches, other researchers within Iran and in other countries in this region can present new figures about C to compare their findings.

## 5. Conclusion

In conclusion, our results showed that the active network size of Iranian people is something between 308 and 380, which is greater than that in western countries. It also seems that this number has a direct association with education level, and reverse association with age. In addition, we found that Iranian males had wider social network. Our results can be applied in estimation of different, hard-to-count population sizes.
